# Retrocaval ureter associated with cryptorchidism: A case report and review of literature

**DOI:** 10.1002/ccr3.1670

**Published:** 2018-07-01

**Authors:** Manoj Hilary Fernando, Umesh Jayarajah, Arulprashanth Arulanantham, Serozsha Goonewardena, Manjula Wijewardena

**Affiliations:** ^1^ Department of Urology National Hospital of Sri Lanka Colombo Sri Lanka

**Keywords:** circumcaval ureter, cryptorchidism, pyelo‐pyelotomy, retrocaval ureter, uretero‐ureterotomy, venous anomaly

## Abstract

We report an incidentally diagnosed retrocaval ureter in a 14‐year‐old boy, while evaluating for right‐sided hydronephrosis associated with cryptorchidism. Therefore, we suggest that any significant right‐sided hydronephrosis associated with cryptorchidism may require investigations to exclude this rare anomaly.

## 1 INTRODUCTION

Retrocaval ureter is a rare condition due to the anomalous development of the inferior vena cava (IVC).^1^ In this condition, the proximal ureter passes posterior to the IVC at the level of the third lumbar vertebra to lie at its medial aspect while the distal part returns to its normal position crossing the IVC from medial to lateral aspect anterior to the lower part of the IVC. Obstruction is usually at the retrocaval segment of the ureter as it lies compressed between the IVC and the body of the third lumbar vertebra.^1^ Bateson Atkinson type 1 retrocaval ureter is the commoner anomaly. This usually presents during the 3rd to 4th decades of life. Pediatric presentation is rarer, and only a few cases were reported. Patients present with symptoms of flank pain, hematuria, and recurrent pyelonephritis with or without secondary urolithiasis.^2^, ^3^, ^4^ Although several other developmental anomalies have been implicated with this condition, the association with cryptorchidism is unusual. We report an incidentally diagnosed retrocaval ureter in a young boy associated with cryptorchidism.

## 2 CASE SUMMARY

A 14‐year‐old boy was investigated for an undescended left testis. He was from a poor socioeconomic background with parental neglect. He was otherwise clinically asymptomatic. The clinical examination revealed a hypoplastic left hemiscrotum with an impalpable left testis and a normal right testis. He did not have hypospadias or other stigmata of developmental anomalies. Ultrasound scan detected a possible atrophic intra‐abdominal testis on the left side and moderate to gross hydronephrosis of the right kidney with proximal hydroureter. X‐ray KUB (kidney‐ureter‐bladder) was normal. Laparoscopic exploration was performed by the general surgical team which confirmed an atrophic intra‐abdominal testis on the left side. A laparoscopic left orchidectomy was performed, and he was referred to the urology unit for further assessment of hydronephroureter.

**Figure 1:**
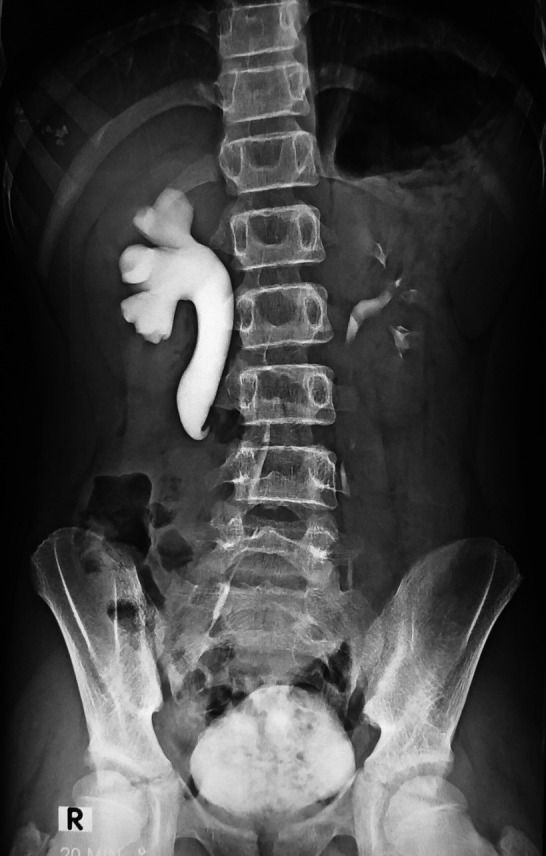
Intravenous urogram showing the pathognomonic “fish‐hook” or the “S shaped” contrast illumination of the proximal ureter

Intravenous urogram (IVU) was performed which revealed a moderate hydronephrosis with proximal hydroureter up to the transverse process of the third lumbar vertebra on the right side with a classical “S‐shaped” or the “fish hook” deformity with faint contrast illumination at the distal ureter which was pathognomonic of retrocaval ureter (Figure 1).^5^ Furthermore, calyceal blunting was also noted. Although this was incidental, considering the significant obstructive uropathy and socioeconomic background, he was offered an open retroperitoneal transposition pyelo‐pyelotomy (uretero‐ureterostomy) to rectify the anomalous retrocaval ureter.

**Figure 2:**
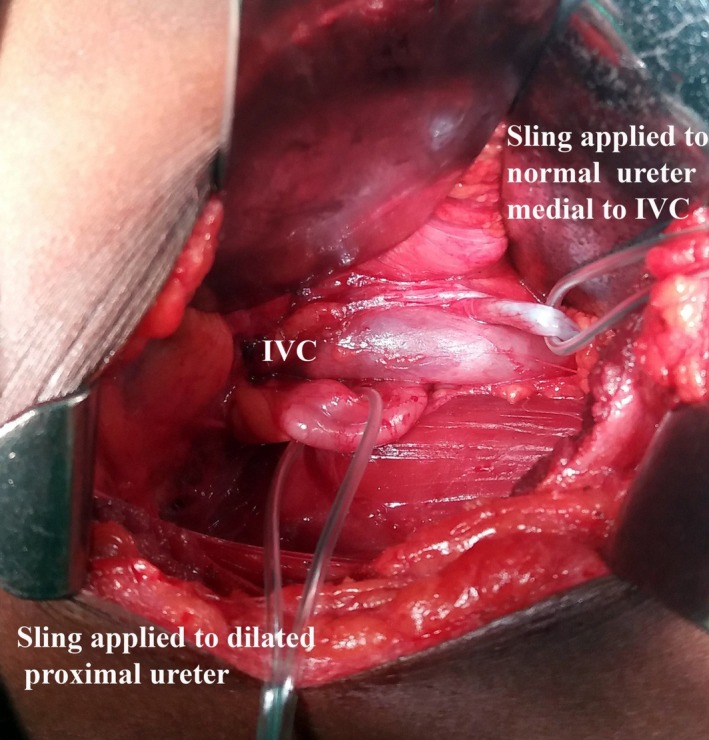
Figure showing mobilized dilated proximal and nondilated medial ureter with the in‐situ JJ stent

A preliminary cystoscopy was performed, and a 5‐Fr JJ stent was positioned over a hydrophilic guide wire to assist easy identification and mobilization of the ureter. A retroperitoneal approach through a 5‐cm muscle cutting flank incision was adopted. Dilated proximal ureter, IVC, and the normal caliber medial ureteric segment were identified and mobilized. Tapes were applied to the ureter besides the IVC to demonstrate retrocaval ureter (Figure 2). Proximal dilated ureter was dismembered in oblique fashion lateral to the IVC, and segment behind the IVC was mobilized and transpositioned anterior to the IVC. Careful examination was carried out to look for any unhealthy stenosed segment of the ureter and to identify concomitant venous abnormalities. Our patient had a healthy distal ureter and did not require excision of a segment prior to spatulated anastomosis over a 5‐Fr stent. The postoperative recovery was uneventful. Technetium 99 m diuretic renography performed at 12 weeks postsurgery revealed satisfactory drainage (Table 1).

**Table 1:** Summary of results of the technetium 99 m diuretic renography

Parameters	Left	Right	Total
Split function (%)	43.5	56.5	100
Kidney counts (cpm)	45 719	59 454	‐
Kidney depth (cm)	3.978	4.003	‐
Uptake (%)	4.569	5.941	10.5
GFR (mL/min)	41.9	54.4	96.3
Normalized GFR (mL/min)	‐	‐	130.2
Mean GFR (mL/min)	‐	‐	118.0
Time of max (min)	2.251	2.251	‐
Time of half max (min)	8.352	10.6	‐

## 3 DISCUSSION

Retrocaval ureter is a rare congenital anomaly due to the abnormal development of IVC. Failure of the right posterior cardinal vein to atrophy in the lumbar region results in this uncommon condition.^1^ Retrocaval ureter is generally right‐sided, unless situs inversus or duplication of the IVC intervenes. Symptoms usually present in the 3rd to 4th decades of life as a result of progressive obstructive uropathy. Rarer pediatric presentations are usually symptomatic similar to adults.^2^, ^3^, ^4^

Retrocaval ureter was first documented in 1893 by Hochestetter on an autopsy study.^6^ Incidence in autopsy studies is about 1:1500, and males were affected three to four times compared to females. The first recorded clinical case by Anderson and Hynes in 1949 described a symptomatic 10‐year‐old girl treated with a reconstructive procedure the “Anderson‐Hynes Pyeloplasty”.^6^

From an embryological point of view, retrocaval ureter is an anomalous segmental development of IVC rather than a ureteric anomaly. Normal abdominal IVC that lies medial to the ureter is formed by the anastomosis of the right vitelline vein forming the hepatic segment, the right subcardinal vein forming the renal segment, and the right sacrocardinal vein giving rise to the sacrocardinal segment of the IVC, between 6th and 8th weeks of gestation. Primitive right posterior cardinal vein that lies lateral to the developing ureter usually atrophies by this time. Abnormal persistence of the right posterior cardinal vein contributing to the formation of the IVC leads to this unusual anomaly.^1^ An embryological relationship between retrocaval ureter and cryptorchidism has not been previously described, and thus, this could be an area of future research interest.

Symptoms occur exclusively in type 1 retrocaval ureter as a result of progressive upper urinary tract obstruction. These include right flank pain, hematuria, recurrent pyelonephritis, and secondary urolithiasis.^2^, ^3^, ^4^ The majority of reported pediatric cases are symptomatic and have a clinical picture similar to adults. Persistent microscopic hematuria was seen in one of these patients.^2^, ^3^, ^7^ A few pediatric cases were diagnosed incidentally, and subsequent surgical correction was performed due to advanced upper tract dilatation secondary to ureteric obstruction.^4^ Although our patient was asymptomatic at presentation, surgery was offered considering the degree of hydronephrosis, calyceal blunting and lack of social support for reliable close follow up to monitor progressive renal impairment. Renal loss has been reported in long‐standing neglect of progressive obstruction in patients with retrocaval ureters.^8^

In 21% of cases, retrocaval ureter was known to be associated with other congenital anomalies, mainly cardiovascular such as duplicated IVC and genitourinary, including horseshoe kidney, contralateral renal hypoplasia or ectopia, congenital absence of vas deferens, and hypospadias.^9^ Furthermore, conditions such as Turner syndrome, Goldenhar syndrome, retroperitoneal fibrosis, and polycystic kidney disease are also associated with retrocaval ureter.^8^ Moreover, supernumerary lumbar vertebra and syndactyly and, in another instance, intestinal malrotation were reported in association with retrocaval ureter.^9^, ^10^

To date, cryptorchidism has not been reported to be associated with this rare condition in the literature. In our patient, investigation of left undescended testis led to the early diagnosis of retrocaval ureter. Therefore, a possibility of a retrocaval ureter should be considered in hydronephrosis associated with cryptorchidism. However, when hydronephrosis coexists with cryptorchidism, other more common congenital abnormities should be considered first, which include pelviureteric junction obstruction and horseshoe kidney. Therefore, an IVU should be performed to evaluate the causes of hydronephrosis.

## 4 CONCLUSION

We reported a 14‐year‐old male with a right‐sided, type 1 retrocaval ureter associated with cryptorchidism. Although several developmental anomalies have been implicated with retrocaval ureter, the association with cryptorchidism is unusual. We suggest to evaluate incidental hydronephrosis associated with cryptorchidism to look for this rare developmental anomaly. However, when hydronephrosis coexists with cryptorchidism, other more common congenital abnormities should be considered first, such as pelviureteric junction obstruction and horseshoe kidney. An IVU should be performed to evaluate the causes of hydronephrosis.

## CONFLICT OF INTEREST

Authors declared there are no conflict of interest.

## CONSENT

Informed written consent was obtained from the patient and the guardian prior to collection of the details.

## AUTHORSHIP

HF, UJ, and AA: contributed to collection of information and writing of the manuscript. SG and MW: contributed to the final approval of the manuscript. All authors read and approved the final version of the manuscript.
